# QTL Analysis of Five Morpho-Physiological Traits in Bread Wheat Using Two Mapping Populations Derived from Common Parents

**DOI:** 10.3390/genes12040604

**Published:** 2021-04-20

**Authors:** Paolo Vitale, Fabio Fania, Salvatore Esposito, Ivano Pecorella, Nicola Pecchioni, Samuela Palombieri, Francesco Sestili, Domenico Lafiandra, Francesca Taranto, Pasquale De Vita

**Affiliations:** 1 Department of Agriculture, Food, Natural Science, Engineering, University of Foggia, Via Napoli 25, 71122 Foggia, Italy; paolo.vitale@unifg.it (P.V.); fabio.fania@unifg.it (F.F.); 2 Research Centre for Cereal and Industrial Crops (CREA-CI), CREA—Council for Agricultural Research and Economics, 71122 Foggia, Italy; salvatore.esposito@crea.gov.it (S.E.); ivano.pecorella@crea.gov.it (I.P.); nicola.pecchioni@crea.gov.it (N.P.); 3 Department of Agriculture and Forest Sciences (DAFNE), University of Tuscia, 01100 Viterbo, Italy; palombieri@unitus.it (S.P.); francescosestili@unitus.it (F.S.); lafiandr@unitus.it (D.L.); 4 Institute of Biosciences and Bioresources (CNR-IBBR), 80055 Portici, Italy

**Keywords:** bread wheat, SNP markers, genetic map, QTL, RILs, F_2_

## Abstract

Traits such as plant height (PH), juvenile growth habit (GH), heading date (HD), and tiller number are important for both increasing yield potential and improving crop adaptation to climate change. In the present study, these traits were investigated by using the same bi-parental population at early (F_2_ and F_2_-derived F_3_ families) and late (F_6_ and F_7_, recombinant inbred lines, RILs) generations to detect quantitative trait loci (QTLs) and search for candidate genes. A total of 176 and 178 lines were genotyped by the wheat Illumina 25K Infinium SNP array. The two genetic maps spanned 2486.97 cM and 3732.84 cM in length, for the F_2_ and RILs, respectively. QTLs explaining the highest phenotypic variation were found on chromosomes 2B, 2D, 5A, and 7D for HD and GH, whereas those for PH were found on chromosomes 4B and 4D. Several QTL detected in the early generations (i.e., PH and tiller number) were not detected in the late generations as they were due to dominance effects. Some of the identified QTLs co-mapped to well-known adaptive genes (i.e., *Ppd-1*, *Vrn-1,* and *Rht-1*). Other putative candidate genes were identified for each trait, of which *PINE1* and *PIF4* may be considered new for GH and TTN in wheat. The use of a large F_2_ mapping population combined with NGS-based genotyping techniques could improve map resolution and allow closer QTL tagging.

## 1. Introduction

Bread wheat (*Triticum aestivum* L.) is grown on more than 200 million hectares of land worldwide, and in 2020, the global production reached about 760 million ton [1]. Despite this, by 2050, wheat production may need to be increased by at least 50% relative to current levels [2,3]. Although crop yields continue to increase globally, climate change represents a tremendous challenge for achieving this objective. In fact, recent research suggests that some major crop yields have already stagnated or even been reduced by the impact of climate change [4]. Phenology genes also regulate the physiological development of wheat [5], and some morpho-physiological traits have been identified as effective in breeding drought-adaptive varieties [6]. Traits such as flowering time, plant height, tiller number, growth habit, flag leaf angle, and spike characteristics, which are important for increasing crop yield potential, are also functional in determining the adaptation of wheat to climate change [7].

Genetic improvement has contributed significantly to modify wheat architecture by reducing plant height through selection of specific alleles at *Rht-1* loci [8] as well as by adjusting flowering time. It is well known that *Ppd* genes influence the plant height, by regulating the number of leaves emitted during the vegetative phase of wheat development, the number of tillers, and fewer spikelets per spike [9]. Giunta et al. [10] demonstrated a major role for the *Vrn* genes on the control of tillering, which was independent of any photoperiod or temperature effects. The wheat ideotype generated during the Green Revolution was also characterized by an erect juvenile growth habit, a reduced number of tillers, and a reduced flag leaf angle [11]. If, on the one hand, this made it possible to increase the investment of plants per unit of area, reducing the intraspecific competition, on the other hand, it made the crop more vulnerable to weeds, reducing interspecific competition. Unfortunately, the trade-off between productivity in weed-free situations and competitive ability is a main obstacle to the release of competitive cultivars [7]. Genetic linkage mapping in segregating populations is one of the traditional approaches to gain insights into the genetic control of key characteristics using quantitative trait locus (QTL) analysis and is a suitable method underlying the molecular marker-assisted selection (MAS) in wheat [12]. The commonly used mapping populations in plants include F_2_, backcross (BC), double haploid (DH), and recombinant inbred line (RIL) populations [13,14]. Among these, the F_2_ population, using codominant markers, provides the most abundant genetic information, including effects such as epistasis and dominance [15]. Ferreira et al. [16], in a simulation study, showed that more accurate maps were obtained with F_2_ codominant and RILs than with BC, DH, and F_2_-dominant populations.

Several studies have been successfully conducted using F_2_ or F_2_-derived F_3_ populations to identify major QTLs in bread wheat [17,18,19], durum wheat [20], rice [21], maize [22], and other species [23,24,25,26]. However, unfortunately, F_2_ and BC are temporary populations. To analyze QTL × E interactions it is necessary to employ permanent populations such as DHs and RILs that can be maintained under several experimental conditions [14].

The RIL populations have several advantages for use in QTL mapping, which have been described by several authors [27,28,29,30]. Multiple selfing processes can increase the number of recombination events [31], which results in a finer mapping of QTLs, but the most important aspect is that once RILs are established they can be repeatedly used for investigating the QTLs of various phenotypes under different environments [10]. However, at least six generations are required to obtain the RILs and they are not suitable to estimate dominance effects of mapped QTLs due to the absence of heterozygous genotypes [32]. Nonetheless, RILs have been extensively used in wheat to identify QTLs for yield [33,34] and other important agronomic traits such as plant height [35], flowering time [36,37,38], and tiller number [39,40,41,42,43]. Otherwise, studies conducted for mapping the juvenile growth habit have been very limited [44].

The knowledge previously acquired by using bi-parental populations and the current availability of high-throughput and cost-effective single nucleotide polymorphism (SNP) genotyping platforms have opened the way for understanding the genetic mechanisms of these important agronomic traits [45].

Stable QTLs are a prerequisite for their use in a marker-assisted breeding program. To validate the QTLs, it is necessary to confirm them in other mapping populations, or in different generations from the same crossing, or using the same RIL population evaluated in multiple locations and in multiple years [46]. QTLs with large effects should be detected consistently across generations, but the increased precision of the RILs should allow the detection of QTLs with smaller effects [16]. In addition, additional opportunities for recombination should improve the genetic resolution of linked QTLs and distinguish linked effects from pleiotropic ones in some instances [47].

In this study, we performed QTL analysis of five morpho-physiological traits, all potentially associated with the yield potential and adaptability in bread wheat, using the same bi-parental population at early (F_2_ and F_2_-derived F_3_) and late (RIL F_6_ and F_7_) stages of inbreeding. We aimed to improve the understanding of the genetic basis (including additive and dominance effect) by comparing the QTLs detected by both populations, which were grown at the same location and evaluated in two years each. In addition, based on a priori knowledge of genes with biological function regulating the investigated traits in wheat and in other species, we also searched for putative candidate genes in chromosome regions tightly linked to QTLs for those traits by exploiting the reference bread wheat genome.

## 2. Materials and Methods

### 2.1. Plant Material and Field Trials

Two mapping populations derived from common parents, Lankaodali and Rebelde, were developed at the Department of Agriculture and Forest Sciences, University of Tuscia, Viterbo, Italy, in collaboration with the CREA Research Centre for Cereal and Industrial Crops (CREA-CI), Foggia, Italy. The female parent, Lankaodali, is a Chinese bread wheat cultivar with very large kernels [48], low tillering capacity, and a medium flowering time. The male parent, Rebelde (Bologna/CH-710//Bologna), is an Italian hard winter wheat cultivar with excellent agronomic characteristics, medium-late flowering time, and high tillering capacity. The two cultivars were crossed for generating first an F_2_ population; then, by advancing through single seed descent, a F_6_ RIL population was generated.

A total of 176 F_2_ individual plants and F_2_-derived F_3_ families of 15 plants (hereinafter referred to as F_3_) together with the parents were grown in a randomized block design with two replications during the two consecutive growing season 2014–2015 and 2015–2016 at CREA-CI (41°27′40.2″ N 15°30′04.5″ E). F_2_ and F_3_ seeds were individually hand-planted in single rows, 2 m length, and 0.5 m between rows.

The RIL population comprising 178 lines and the two parents were planted during the two consecutive growing seasons 2017–2018 and 2018–2019 (hereinafter referred to as F_6_ and F_7_, respectively) at the same experimental site. To simulate a standard open field sowing, a standard sowing density corresponding to approximately 350 seeds per square meter was carried out in single rows, 2 m long and 0.20 spaced, in a randomized block design with two replications. Whole plants of the F_2_, F_3_, and the two RIL generations (F_6_ and F_7_) were manually harvested at the end of June 2015, 2016, 2018 and 2019, respectively. Crop management was performed using standard cultivation methods. Growing season precipitation and temperature data were collected from the meteorological station of the CREA-CI.

### 2.2. Phenotypic Evaluation

Traits were measured both on a row and individual plant basis as follows: Heading date (HD) was recorded as days from the 1st of April in all growing seasons when 50% of the plants in a row were at GS59 [49]. Plant height (PH, cm) of individual F_2_ and F_3_ plants and ten randomly selected plants from RILs were measured at maturity. The juvenile growth habit trait (GH) was estimated by measuring the tiller angle between the last developed tillers and the ground level with a protractor at the maximum tillering stage (GS25 to GS29) by following UPOV [44] guidelines: 1, erect; 3, semi-erect; 5, intermediate; 7, semi-prostrate; and 9, prostrate. Tillering was monitored throughout the study period. Ten plants randomly selected on each single row were marked to facilitate identification at harvest. This was necessary for the F_6_ and F_7_ RIL generation with dense planting. At maturity, the number of tillers bearing spikes for each plant was counted, together with the number of fertile spikes. The tillering data were used to calculate the maximum number of tillers (TTN) and the number of fertile tillers (FTN). All phenotypic data were processed in the original form without any transformation. Principal component analysis (PCA) was performed using the mean values of all phenotypic data with the function “prcomp” implemented in the *factoextra* v. 1.0.7 library under R environment v. 4.0.2. Pearson correlation coefficients (r) were calculated among traits using the “cor” function and the correlogram was constructed and visualized using the *Corrplot* package v. 0.84 implemented in R v. 4.0.2 [50]. Coefficient of variation was calculated as follows:

CV = Std/M × 100(1)

where ‘‘Std’’ represents the standard deviation and ‘‘M’’ represents the mean for a trait. Broad-sense heritability (H^2^) was estimated as follows:

H^2^ = σg/[σg + (σgy/y) + (σe/τy)] (2)

where σg is genotypic component of variance, σgy is the variance explained by interaction between genotypes and year, σe is the variance of residual effects, τ is the number of the repetitions, and y is the number of the year.

The Shapiro–Wilk (w) statistics was used to test the null hypothesis that the phenotypic data were normally distributed [51].

### 2.3. Linkage Maps Construction

Plant materials from F_2_, F_6_ generations, and both parents were genotyped with the wheat Illumina 25K Infinium single nucleotide polymorphism (SNP) array that was developed at the Trait Genetics (available online: http://www.traitgenetics.com, accessed on 24 June 2020). Both datasets were filtered by removing missing data (call rate > 10%) and rare alleles (MAF < 1%). Markers showing significant (*p* < 0.01) segregation distortion were also discarded.

The two filtered datasets were used to construct the genetic maps (hereinafter referred to as F_2_ and RIL map) using the package *ASMap* v. 0.4.1 [52]. Linkage groups were constructed using the MSTmap algorithm [53] based on the *p*-value threshold set to 1 × 10^−10^ and 1 × 10^−12^ for F_2_ and RIL populations, respectively. The Kosambi function was used to calculate the distance between markers and convert the recombination frequency into map distance (cM). The recombination fraction between all pairs of markers was estimated by the “est.rf” function to verify if each marker was placed in the right chromosome and in the correct order as compared with the Wen’s reference map [54]. According to the Wen’s reference map, some chromosomes were flipped through the “flip.order” function, whereas different linkage groups belonging to the same chromosome were merged using “mergeCross” function. The colinearity between the F_2_ and F_6_ linkage maps was evaluated by the Spearman rank correlation coefficient calculated by the R function “cor.test”. A comparison between the obtained maps was performed by the R package *SOFIA* v. 1.0 [55].

### 2.4. QTL Mapping Approaches

The QTLs were detected for each trait in both generations by performing genome-wide composite interval mapping (gCIM), using the R software *QTL.gCIMapping.GUI* v.2.0 [56]. The first step was the binning of the redundant markers, as these markers have the same segregation in the population and bring no additional information to QTL analysis. The selected model was fixed and a walk speed for genome-wide scanning set to 1 cM. The LOD score threshold was set to 3.0 for both F_2_:F_3_ and RILs. A maximum likelihood (ML) function was used, the random seeds were set to 11,001, and the completing CIM in one neighborhood was set to “FALSE” for the F_2_:F_3_ population only. Moreover, the effects of QTLs, which include additive and dominance effects, were estimated by empirical Bayesian methods. The QTLs were named based on the recommended rules for gene symbolization in wheat (available online at https://wheat.pw.usda.gov/ggpages/wgc/98/Intro.htm, accessed on 15 January 2021).

### 2.5. Identification of Putative Candidate Genes

Putative candidate genes were identified within the QTL confidence intervals based on the annotation of the Chinese Spring genome, according to the IWGSC RefSeq v1.0 [57] (https://wheat-urgi.versailles.inra.fr/Seq-Repository/Annotation, accessed on 1 February 2021). The acronym of genes was assigned using the UNIPROT database (https://www.uniprot.org/, accessed on 1 February 2021). The sequence of the *PINE1* (LOC_Os12g42250) gene isolated in rice was retrieved by Gómez-Ariza et al. [58], whereas *PIF4* (LOC_Os12g41650) sequence was searched on the rice genome database available at https://shigen.nig.ac.jp/rice/oryzabase/ (accessed on 10 February 2021). The rice nucleotide sequences were used for a TBLASTN search against the genomes of the durum cultivar Svevo (http://www.gramene.org/, accessed on 10 February 2021). Then, the physical map position of the best sequence hits was compared to that of the genes identified in the QTL intervals, to investigate the correspondence.

Finally, all genes found in the QTL intervals were associated with a metabolic pathway by trait using the software MAPMAN [59].

## 3. Results

### 3.1. Phenotypic Evaluation and Correlation Analysis between Early and Late Generations

Mean values, ranges, coefficient of variation (CV), and broad-sense heritability (H^2^) of the investigated traits were calculated for each of the four generations (Table 1). Significant differences between the mean values of the parents were observed in all generations for HD, GH, TTN, and FTN, except for PH in the late generations.

The mean values of HD were higher in F_2_ and F_3_ than in F_6_ and F_7_, while the opposite was observed for PH. F_2_ had the lowest mean value for GH, F_2_ and F_6_ showed the highest values in TTN and FTN. For the five traits, the CV ranged from 10.13 (HD) to 71.94% (GH) in the early generations and from 14.74 (HD) to 37.93% (FTN) in the late generations. The H^2^ values ranged from 0.17 to 0.76 and from 0.37 to 0.83 in the early and in the late generations, respectively. The H^2^ values higher than 0.69 were observed for HD and PH in early and RIL generations, while GH had H^2^ higher in the RILs (0.82) than in the F_2_/F_3_ (0.30). The heritability of the TTN and FTN was low in both generations with the lowest value in the early generations (0.17 and 0.20 for TTN and FTN, respectively).

The Shapiro–Wilk test confirmed the normality of PH, TTN, and FTN data in F_2_ and F_7_ populations, with a right-skewed distribution for the last two traits. In addition, normality was also observed for the trait FTN in the F_3_ and for the traits, GH and PH, in the F_6_ generations. A bimodal distribution for HD was observed in early generations (F_2_ and F_3_) (Supplementary Figure S1).

**Figure 1:**
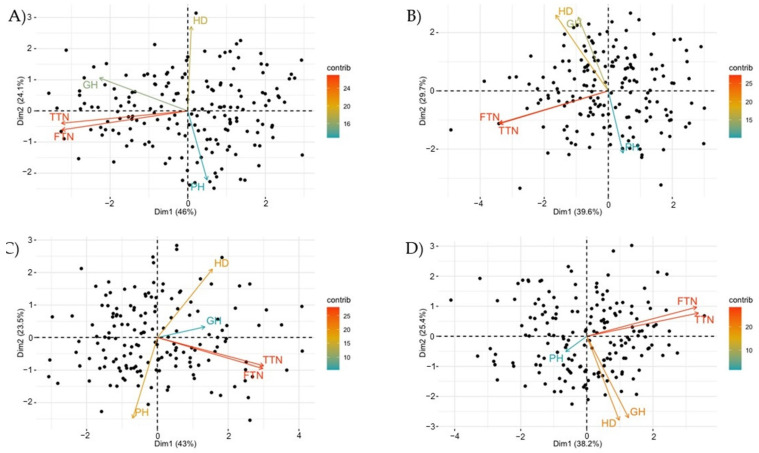
Principal component analysis (PCA) diagram showing the phenotypic variation and variable contribution in F_2_ (**A**), F_3_ (**B**), F_6_ (**C**), and F_7_ (**D**) generations. Black points correspond to genotypes, whereas the arrows represent the contribution of the variables to the total variation. HD, heading date; PH, plant height; GH, growth habit; TTN, total tiller number; FTN, fertile tiller number; contrib, contribution of variables to the principal axes expressed as a percentage (%).

The PCA analysis, based on the five morpho-physiological traits, showed a large variability within the four populations (Figure 1). The first two dimensions explained 70.1%, 69.9%, 66.5%, and 63.6% of the total variance in the F_2_, F_3_, F_6_, and F_7_ generations, respectively.

The first component discriminated PH from the other traits in all populations, except for the HD in F_2_. The second dimension discriminated HD and GH from PH, FTN, and TTN in all generations except for F_7_.

Finally, Supplementary Figure S2 and Table S2 show that in the four generations, a significant positive correlation was observed between TTN and FTN (r > 0.82 and *p*-value < 1.33 × 10^−44^), between GH and HD in F_3_ (r = 0.40, *p*-value = 2.79 × 10^−8^) and F_7_ (r = 0.33, *p*-value = 7.55 × 10^−6^), and between GH and TTN/FTN in F_2_ (r = 0.45, *p*-value = 4.32 × 10^−10^ and r = 0.45, *p*-value = 4.66 × 10^−10^), F_6_ (r = 0.19, *p*-value = 1.40 × 10^−2^ and r = 0.21, *p*-value = 9.16 × 10^−3^), and F_7_ (r = 0.15, *p*-value = 5.13 × 10^−2^ and r = 0.11, *p*-value = 0.15). By contrast, a significant negative correlation was observed between PH and HD in F_3_ (r = −0.28, *p*-value = 2.13 × 10^−3^) and F_6_ (r = −0.27, *p*-value = 7.55 × 10^−6^).

### 3.2. Construction of F_2_ and F_6_ Linkage Maps and Their Colinearity

After filtering, 3684 and 3958 SNP variants were retained for F_2_ and F_6_, respectively, and used to build the two genetic maps. Thirty-nine and thirty-eight linkage groups were identified in F_2_ and F_6_ populations, respectively. The smaller linkage groups were joined to form the twenty-one chromosomes of *T. aestivum* L., based on the information available in the consensus map of Wen et al. [54]. The two genetic maps spanned 2486.97 and 3732.84 cM in length, for the F_2_ and RILs, respectively, with an average marker density of 1.37 and 1 marker/cM (Supplementary Table S3).

The coverage was 47.49%, 36.82% and 15.69% for genome A, B, and D, respectively, in the F_2_, and 46.58%, 42.28%, and 11.14% in the F_6_.

**Figure 2:**
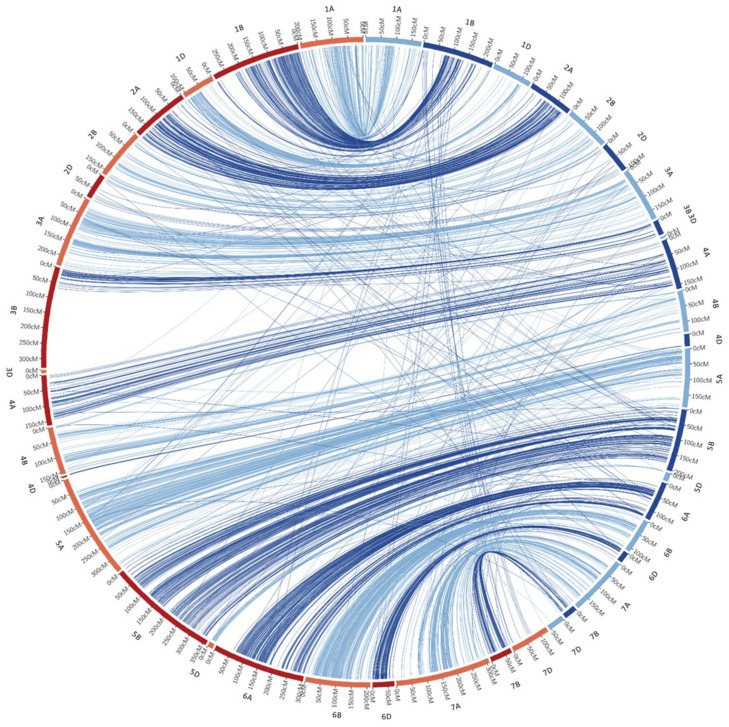
Circle plot showing the collinearity between the 21 linkage groups generated by F_2_ (blue lines, right side) and F_6_ (red lines, left side) populations.

The chromosomes length ranged from 5.01 (chr. 3D) to 231.56 cM (chr. 1B) with an average length of 118.43 cM in the F_2_ map (Supplementary Table S4). Among the RILs, the chromosomes length varied between 3.93 (chr. 4D) and 360.26 cM (chr. 5B), with an average of 177.75 cM. The distance between adjacent markers for each chromosome ranged between 0.26 (chr. 3D) and 5.50 cM (chr. 7D) with an average distance of 1.15 cM in the F_2_ map, and between 0.55 (chr. 2A) and 7.96 cM (chr. 7D) in the F_6_, with an average of 1.50 marker/cM. The collinearity was calculated between the two genetic maps (Figure 2 and Supplementary Table S4). The Spearman rank correlation coefficient of each chromosome was calculated for the F_2_ and F_6_ linkage maps, ranging from −1 (chr. 7D) to 0.95 (chr. 1A) (Supplementary Table S4). Among all the twenty Spearman rank correlation coefficients, six were higher than 0.90 (chr. 1A, 2A, 3A, 5B, 6B, and 7A), and seven were comprised between 0.80 and 0.89 (1B, 2B, 4B, 5A, 6A, 6D, and 7B). Three chromosomes on D genome (2, 4, and 7) showed a negative correlation.

### 3.3. QTL Identification for Different Traits

**Figure 3:**
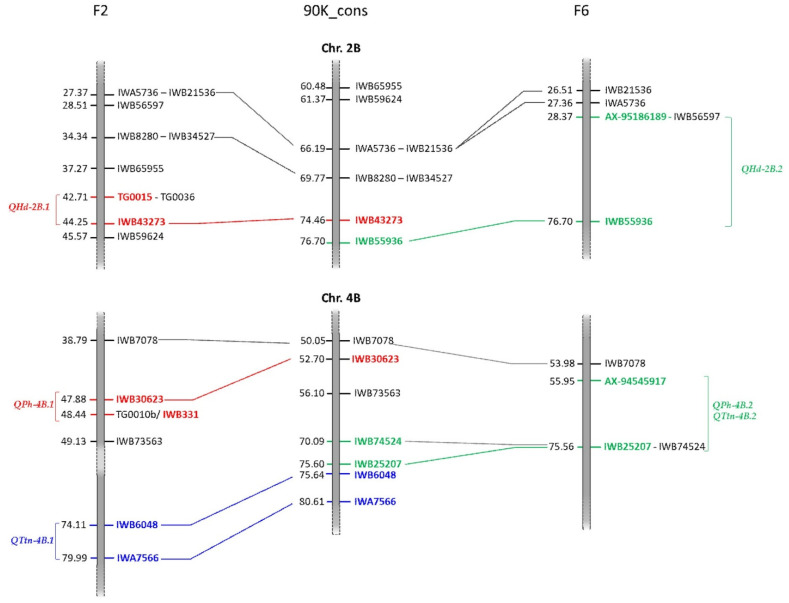
A graphical representation of the 2B and 4B linkage groups derived from the F_2_ and F_6_ maps and their comparison with the 90K consensus map. The distance between markers is in centimorgans (cM). The markers flanking the QTLs are colored in red (*QHd-2B.1* and *QPh-4B.1*) and blue (*QTtn-4B.1*) in F_2_, while in green for the QTLs (*QHd-2B.2*, *QPh-4B.2,* and *QTtn-4B.2*) in F_6_. Solid lines connect the genetic loci in common between the F_2_ and F_6_ genetic maps and the 90K consensus map [60].

A total of 37 QTLs associated with the five morpho-physiological traits were detected using all datasets separately (F_2_, F_3_, F_6_, and F_7_), of which 28 and 10 were detected in the early (F_2_ and F_3_) and late (F_6_–F_7_) generations, respectively (Table 2). One QTL (*QGh-2D.1*) was identified both in F_3_ and F_7_, whereas three QTLs were identified in overlapped regions between F_2_/F_3_ and F_6_/F_7_ (Figure 3). The QTLs were distributed across all chromosomes except for 1D, 5D, 6A, 6B, 6D, and 7B. Twelve, sixteen and nine QTLs were mapped to the A, B, and D genomes, respectively. The phenotypic variation explained by an individual QTL was estimated between 0.53 (*QTtn-1A.1*) and 30.98% (*QHd-5A*). The LOD values ranged from 3.04 (*QFtn-4B*) to 18.78 (*QHd-5A*).

For HD, six QTLs were identified on chromosomes 2B, 2D, 4D, 5A, and 7D, with individual QTL explaining 3.70–30.98% of the phenotypic variance. Of these, two QTLs were detected on chromosome 5A (*QHd-5A*) and 7D (*QHd-7D*), both in F_6_ and F_7_ populations, with individual QTLs explaining the highest phenotypic variance in the F_7_ (R^2^ = 30.98 and 16.68% for *QHd-5A* and *QHd-7D*, respectively). Two distinct QTLs (*QHd-2B.1* and *QHd-2B.2*) were found on chromosome 2B in the F_2_ and RILs. However, the markers flanking the two QTLs were adjacent each other, with a distance of 2.24 cM according to the 90K consensus map [60] (Figure 3). One QTL (*QHd-4D*) was identified on chromosome 4D both in F_2_ and F_3_, exhibiting 1.82% and 6.10% of the phenotypic variance, respectively. One QTL (*QHd-2D*) was identified on chromosome 2D in F_2_, with a negative additive effect.

Six QTLs were found for GH in both mapping populations (Table 2). Among all regions, a major QTL was found on chromosome 5A in late generations (F_6_ and F_7_), explaining the highest LOD scores and phenotypic variance (LOD = 7.23 and 10.21, R^2^ = 13.1% and 22.6% in F_6_ and F_7_, respectively). On chromosome 1B, two adjacent regions were identified explaining a high fraction of phenotypic variance (12.37% and 9.31% for F_6_ and F_7_, respectively). Two QTLs were identified on chromosome 2D, of which, *QGh-2D.1* was detected both in F_3_ and F_6_, while the *QGh-2D.2* was mapped only in F_6_. Another QTL (*QGh-4B*) was found on chromosome 4B in F_3_, with a dominance effect of −0.67.

A total of five QTLs were found associated with PH. Among all, the QTLs on chromosome 4B (*QPh-4B.1* and *QPh-4B.2*) explained a high proportion of phenotypic variation on F_2_ (16.39%), F_6_ (26.41%), and F_7_ (21%). Although the markers flanking the *QPh-4B.1* and *QPh-4B.2* were different, the two regions could be considered overlapping, as showed in Figure 3. In fact, although many markers were not in common between the two genetic maps in this region, IWB7078 was the closest marker to *QPh-4B.1* and *QPh-4B.2.* On early generations, a significant QTL was mapped on chromosome 4D (*QPh-4D*). Another two QTLs (*QPh-2D* and *QPh-5A*) were identified, explaining 2.28 and 1.79% phenotypic variation, respectively.

The highest number of QTLs were detected for TTN. Seventeen QTLs were identified and distributed on almost all chromosomes (1A, 1B, 2B, 3A, 3D, 4A, 4B, 4D, 5A, 5B, 7A, and 7B). Among these, nine were identified by a single marker. All QTLs were detected in F_2_ population, except for *QTtn-4B.2* (F_6_), which showed the highest explained phenotypic variance (10.49%). The regions identified from the two QTLs, *QTtn-4B.1* and *QTtn-4B.2,* could be considered to be overlapping between F_2_ and F_6_ maps (Figure 3).

For FTN, three QTLs were found on the chromosomes 1B, 4B, and 7A in the early generations. Some QTLs showed a lower level of phenotypic variance ranging from 4.26% (*QFtn-7A*) to 2.67% (*QFtn-1B*).

Finally, we found that QTLs for different traits were located in the same chromosomal region. The flanking markers AX-94545917-IWB25207 identified a common region on chromosome 4B (*QPh-4B.2* and *QTtn-4B.2*) in late generations; the marker IWA6294 was associated with the *QTtn-1B.1* and *QFtn-1B* on chromosome 1B in F_2_; the markers IWA6802-IWA3719 flanking the *QTtn-7A.2* and *QFtn-7A*; the *QHd-5A* and *QGh-5A* were delimited from the markers IWA6961-AX-94636029, while *QHd-2D* and *QPh-2D* from IWB3771-IWB7001 markers. The *QHd-4D* and *QTtn-4D* were flanking from IWB61486-IWB30224 markers.

### 3.4. Candidate Genes and Their Metabolic Pathway

Several genes modulating photoperiodic flowering pathway were annotated within QTLs (Table 2 and Supplementary File S1). Two transcriptor factors (TF) involved in flowering control, the *zinc-finger premature internode elongation 1* (*PINE1*, TraesCS4B01G342300) and the *phytocrome-interacting bHLH factor* (*PIF4*, TraesCS5A02G04960), were identified within QGh-4B and QTtn-5A, respectively. In addition, TFs that play a role in the regulation of flowering time and tillering, such as *DELLA protein gai* (*GAI*), *flowering-promoting factor* (*RAA1*), *Ap2-like ethylene-responsive* (*AP2/ERF*), *growth-regulating factor* (*GRF*), *ethylene-responsive* (*ERF*), *zinc finger protein constans* (*CO*), *Gras* (*SLC*), *AGAMOUS-MADS-box* (*AGL*), *zinc finger CCCH domain-containing* (*ZC3H*), and *NAC* domain were also annotated. We also identified genes that function in hormonal controlled steps of development, such as *gibberellin 2-beta-dioxygenase 2* (*GA2OX2*), *gibberel-lin 3-beta-dioxygenase 2* (*GA3OX2*), *gibberellin-regulated protein* (*GASA1*), *auxin response factor* (*ARF*), *auxin responsive SAUR* (*SAUR*), *flavin-containing monooxygenase* (*YUCCA*), *CLAVATA3/ESR* (*CLE*), and *tornado* (*TRN1*). The following genes, encoding sucrose and starch metabolisms and acting as a signal to control growth and differentiation, were identified: *trehalose-6-phosphate synthase 1* (*TPS6*), sugar transporters (*SWEET*), *sucrose synthase* (*SUS1*), *ribulose bisphosphate carboxylase* (*RUBISCO*), *alpha-amylase* (*AMY*), and *protein targeting to starch* (*PTST*). Finally, genes related to nitrogen metabolism, such as *nitrate reductase* (*NR*) and *nitrate transporter* (*NPF*), and seed storage (*γ-gliadin* and glutenin genes) were detected.

Supplementary Figure S3 summarizes the metabolic pathways in which the candidate genes are involved. Six different pathways were identified for HD, GH, PH, TTN, i.e., photosynthesis, secondary metabolism, tetrapyrrole, carbohydrate, lipid, and N-metabolism. The functional categories more represented for all traits were classified as fermentations and light reactions for photosynthesis, OPP, and the nitrogen assimilation (Supplementary Figure S3).

## 4. Discussion

Traits such as plant height, heading date, juvenile growth habit, and tiller number, historically, have been subjected to strong selective natural and artificial pressure, to improve the adaptation of bread wheat to different climatic conditions and to increase the grain yield [66,67,68,69]. However, these same traits are not only important for increasing crop yield potential, but they are also useful in determining the adaptation to climate change [7].

In the present work, the genetic control of five morpho-physiological traits was investigated, using two mapping populations derived from the same parents, to identify associated QTLs and candidate genes.

To achieve our aims, plants were deep phenotyped during four growing seasons. High heritability was recorded for PH and HD in both mapping populations. This was consistent with previous reports in wheat and also in other plant cereal species such as rice and barley, indicating a high response to selection of these traits [70,71,72,73]. By contrast, low heritability was found for GH, TTN, and FTN, as also recently reported by Marone et al. [44] and Bilgrami et al. [74] for juvenile growth habit and tiller number, respectively. Continuous distribution or absence of discrete segregating classes for PH, TTN, and FTN suggested that its inheritance is either determined by a large number of genes with small effects or by a few major genes with substantial environmental effects. The presence of transgressive segregants in all traits investigated suggested that each of the two parental cultivars had desirable and undesirable alleles in various proportions for loci governing these traits, as shown in Table 2.

More than 3000 high quality SNP markers were used to build the two genetic maps, and as expected, most of them were placed on genome A and B, in line with previous results [75,76,77]. Wen et al. [54] showed that the D genome had fewer markers than the A and B genomes in the high-density consensus map in common wheat.

The resulting maps were different in length, being 2486.97 cM in the F_2_ and 3732.84 cM in the F_6_. Similar to our results, Price and Tomos [78] obtained a map length of roughly 1280 cM in the F_2_ population in rice, whereas a longer one (1680 cM) was observed in the F_6_ produced by self-pollination of the same F_2_ [79]. The observed differences were probably due to the different levels of heterozygosity, ranging about 50% and 5% in F_2_ and F_6_ RIL generations, respectively.

A total of 28 and 10 QTLs were found in the F_2_/F_3_ and RIL populations, respectively, for all traits (Table 2). Among all QTLs identified in the present study, only one was detected across both generations. However, the comparative QTL analysis of chromosomes 2B and 4B between F_2_ and F_6_ populations showed that three QTLs for HD, PH, and TTN could be considered to be adjacent and nearly overlapping. The results of the joint analysis indicate F_2_/F_3_ progenies have a higher power for detecting QTLs than the RIL populations. This is probably because the QTLs with dominant effects were only detected in the F_2_:F_3_ but not in the RIL generations. For quantitative traits with low heritability, such as TTN and FTN, the QTLs were identified only in early generations. This contrasted with what was expected, given that for quantitative traits, the precision of QTL mapping with an F_2_ population may be relatively poor and, to solve this problem, a corresponding F_3_ progeny was phenotyped, as proposed by Zhang and Xu [80]. However, in our study a possible explanation could be linked to the different sowing method adopted; spaced plantings in early generations allowed a better and more accurate evaluation of the tillering capacity of the wheat lines [81]. Furthermore, a higher marker density was observed in F_2_ than in F_6_, in the QTL regions. The low density of markers could affect the QTL analysis in the RIL populations [12,82]. Indeed, Yi et al. [83], in a previous study, which included QTLs consistency across generations derived from the same cross, suggested that results largely depend on many factors including generations, environment, and genetic backgrounds.

Pearson rank between the assessed traits revealed that HD was correlated with the different morpho-physiological traits such as GH, PH, and TTN, in agreement with Bilgrami et al. [74], Rabbi and Hisam [77], and Mecha et al. [84]. In our study, several QTLs for different traits co-localized on the same chromosome, suggesting that they were not distributed evenly in the wheat genome, but they tended to cluster in particular chromosome regions (Table 2). According to their overlapping intervals, the regions on chromosome 5A, 2D, and 4D contained QTLs that were coincident for HD and GH, HD and PH, as well as HD and TTN, respectively. The two overlapping QTLs for TTN and FTN, found on chromosomes 1B and 7A, such as the common QTL for PH and TTN on chromosome 4B were expected. Other studies have confirmed that several QTLs/genes associated with canopy-architecture-related traits shared regions associated with yield-grain-related traits [85,86,87,88].

According to the physical map information obtained in this study and those of the major genes known in the literature to be responsible for the main morpho-phenolic wheat traits (i.e., *Vrn*, *Ppd,* and *Rht*), several co-localizations were found.

In particular, two overlapped QTLs for HD (*QHd-5A*) and GH (*QGh-5A*) contained the well-known gene *Vrn-A1* (TraesCS5A01G391700). Similarly, *QGh-2D.2* was found close to the photoperiod gene (*Ppd-D1*). This means that adaptation genes such as *Vrn* and *Ppd* influenced the expression of juvenile growth habit trait either because of linkage, or because they exert a pleiotropic effect on this trait, as previously suggested by Marone et al. [44]. As expected, we observed a co-localization among the QTLs *QHd-2B.1* and *QHd-2B.2* and the photoperiod gene *Ppd-B1* [89]. Similarly, the *QHd-2D* was only 4.5 Mb away from the *Ppd-D1* (*TraesCS2D02G079600*) gene. In addition, *QHd-4D* was found to be 7.2 Mb from the *Rht-D1* gene (*TraesCS4D02G040400*), suggesting that genes controlling plant height might also affect flowering time. In general, all the QTLs identified for HD had been extensively reported by the literature [38,61,90,91,92], confirming the strength of the use of two mapping populations in our work.

Similar results were also shown for the PH trait, which is one of the most studied [65]. Two QTLs (*QPh-4B.1* and *QPh-4B.2*) included the well-known dwarfing *Rht-B1* gene (TraesCS4B02G043100) and the region overlapped also with the one recently identified for plant height by Wang et al. [64], and with another QTL associated with maturity date [90]. In addition, the *Rht-D1* gene on chromosome 4D co-localized with the region identified by the *QPh-4D* in both early generations, sharing the common SNP marker RAC875_rep_c105718_304 with Zanke et al. [65].

Regarding TTN, all QTLs were identified in the early generations (with the exception of *QTtn-4B.2* in F_6_) and distributed on almost all chromosomes (Table 2), confirming previous studies conducted in different genetic backgrounds and agronomic contexts [74,93]. *QTtn-4D* was detected very close to *Rht-D1* [65], similar to *QHd-4D*. The QFtn-4B detected on chromosome 4B was also interesting since it covered a genomic region close (6 Mb) to the *Rht-B1* gene. In the past, *Rht-B1b* and *Rht-D1b* were associated with increased productive tillers, which also contributed to increased yield [94,95,96]. Indeed, in our study we found the QTL for TTN in the same regions where the QTL for grain yield (*QTtn-1A.2*) and NDVI at tillering (*QTtn-7B*) were identified [97,98]. In this study a significant association between *Rht* genes and tillering traits was confirmed. The *QTtn-1A.1* identified by the SNP marker IWA6644 has been confirmed by a previous work by Bilgrami et al. [74].

It was observed that favorable alleles for each QTL were contributed by both parents, which was indicated by the additive effect of the QTLs. In addition, QTLs for TTN and FTN in the F_2_ population showed both additive and dominant effects, suggesting the importance of both modes of gene action in conditioning tiller number and the greater difficulty in selection in late generations.

In addition to well-known genes involved in flowering time regulation (*Vrn*, *Ppd*, and *Rht*), QTL mapping detected candidate genes implicated in plant architectural traits including the regulation of the GA pathway, perception of light, photosynthesis, transport of photosynthates, and source-sink relationship. The role of these genes has been widely dissected in species such as rice and *Arabidopsis*, while they are still little investigated in wheat.

Gibberellin is a hormone that plays a central role in plant growth, organ development, and stress responses. In general, the existence of a regulatory network coordinating flowering and GA-dependent growth is well known. In our work we discovered genes coding enzymes of gibberellin biosynthesis (*Ga3ox*, *GA20ox*) and its receptors (DELLA-interacting protein *GID1* and *SD1*) associated with GH and TTN [99,100]. A barley GA 20-oxidase gene (*Hv20ox2*) has been proposed as a candidate for *sdw1/denso*, likely ortholog to the rice *sd1/Os20ox2* gene [101,102,103]. In barley, plants possessing the semi-dwarfing *sdw1/denso* gene have been characterized by prostrate GH, confirming our results [101]. In addition, further analysis revealed that the barley *sdw/denso* gene, has a pleiotropic effect on several agronomic traits such as plant height, heading, and flowering time [104].

Many zinc finger transcription factors have been found on chromosomes (1B, 2D, 4B, 4D, and 7A) such as CCCH-domain zinc finger proteins, which are known to be involved in plant growth habit and in leaf angle regulation [105]. Our findings confirmed the role of these transcription factors in determining the bread wheat GH, as reported in durum wheat [44].

Among its various functions, GA promotes the phenomenon of stem and leaf elongation [99]. In the flowering plants, altering internode length permits plants to vertically shift the distribution of their leaves and buds attached at nodes, changing their growth habit. Benefits of internode elongation at flowering include inflorescence escape from soilborne pests, outcompeting neighbors, and potential for diversified pollination and wider seed dispersal. In our work, the gene *PINE1* was a strong candidate associated with GH. It was recently identified in rice as a transcription factor involved in coordinating internode elongation and photoperiodic signals. It regulates the reduction of the sensitivity of the stem to gibberellin, with a consequent promotion of flowering [58].

In addition, we found a TF belonging to the *NAC* family associated with all traits. The *NAC* genes coding for TF have been shown to influence plant height through regulating the key genes in the GA pathway, as well as flowering time [106]. It has been observed that the *OsNAC2* promotes shoot branching; indeed, its overexpression resulted in more tillers [107]. Transgenic plants that constitutively expressed *OsNAC2* had shorter internodes, shorter spikelets, and were more insensitive to gibberellic acid. In addition, the *ERF* genes that we identified in QTLs related to TTN, trigger the upregulation of the internode elongation, through an increase in bioactive GA levels [108].

Plants alter their morphology to avoid the risk shading, through changes associated with the light spectrum and intensity. The responses to shade avoidance include variation in the hypocotyl and stem and activation of multiple hormones such as auxin, ethylene, and GA. The transcription factor *PIF4*, which we found within QTLs for TTN, is activated by the photoreceptors signal via separate pathways in leaf responses in *A. thaliana*. *PIF4* is a target for GA signaling through interaction with DELLA proteins, but also *GA20ox* and *GA3ox* expression, with a mechanism regulation currently unknown. It has been demonstrated that by destabilizing DELLAs, GA signaling promotes *PIF4* function [99,109].

In addition, during the early growth phase and under abiotic stress conditions, *PIF4* promotes auxin biosynthesis genes such as *YUCCA*, enhancing tissue auxin level that regulates small auxin up-RNA (*SAUR*) genes responsible for hypocotyl elongation when grown under light conditions [110,111,112].

Another important gene is the sucrose-signaling metabolite synthase *trehalose 6-phosphate synthase* (*TPS6*) located in the interval of the *Q.Ttn-3A*, which showed the higher LOD (17.91). This gene encodes what is considered to be a potent signaling molecule in plants for embryogenesis and normal postembryonic growth and development. Wahl et al. [113] indicated that the *TPS6* gene is required for timely initiation of flowering. In *Arabidopsis*, Fichtner et al. [114] suggested that the *TPS6* gene has two different functions depending on some parts of the plant and some developmental stages, both as a signal and as a negative feedback regulator of sucrose levels.

Finally, we found genes involved in nitrogen metabolism and carbohydrate assimilation (*RUBISCO*) and metabolism (*SUS*) associated with all traits. In *Arabidopsis*, it has been demonstrated that there is a significant interaction among nitrate, carbohydrates, *RUBISCO,* and the starch accumulations that affect plant growth and development, stress responses, and signal molecules [111,115,116]. The selected genes underlying the QTL regions represent a partial selection of genes that could regulated the traits investigated, therefore, further studies are needed to validate the causative genes.

## 5. Conclusions

The results of the present study show that QTL mapping results obtained from populations derived from founding parents at different generations are reliable. The exploitation of the two mapping populations made it possible to identify all the main regions of the morphological characters and even identify new regions of interest especially for low heritability characters such as GH ant tiller number. The QTLs detected in early generation are more than those in late generation, especially for those minor QTLs. Further studies are needed to confirm the chromosomal regions responsible for low heritability traits such as TTN and FTN. For these traits, it will be necessary to utilize spaced plantings to properly assess tillering habits of wheat cultivars because plants grown at high population densities in grower production fields are not likely to represent full tillering potential of a line/genotype. It was particularly interesting to observe the strong interaction between the adaptation genes and the morpho-physiological characteristics of the plants. It is evident, in fact, that the genes, in the past, that were fundamental for increasing productivity, will also be so in the near future as they are also responsible for the characters indirectly associated with the new climate scenario. This requires an in-depth study of the effect of allelic variants of adaptation genes by exploiting modern genomic technologies.

## Figures and Tables

**Table 1:** Minimum, maximum, mean, standard deviations (St. Dev), coefficient of variation (CV), and heritability (H^2^) for five traits in the F_2_, F_3_, F_6_, and F_7_ populations. Phenotipic values of both parents (Lankaodali, L and Rebelde, R) are also shown.

Trait	Acronym	Unit	L	R	Min	Max	Mean	St. Dev	CV%	H^2^
Heading Date	HD	Day								
F_2_			16.0	28.0	17.0	30.0	24.36	2.91	11.93	0.74
F_3_			23.0	30.0	22.0	33.0	27.07	2.74	10.13	
F_6_			19.5	25.0	17.0	34.0	23.88	3.50	14.64	0.69
F_7_			13.5	24.0	8.0	30.0	19.98	4.90	24.50	
Juvenile Growth Habit	GH	Scale								
F_2_			1.0	6.0	1.0	7.0	2.69	1.93	71.94	0.30
F_3_			2.0	5.0	1.0	9.0	5.70	1.77	31.10	
F_6_			2.5	5.0	1.4	9.0	5.45	1.42	26.11	0.82
F_7_			2.5	5.5	2.0	9.0	5.74	1.51	26.38	
Plant Height	PH	cm								
F_2_			49.0	75.0	35.0	110.0	66.14	12.68	19.18	0.76
F_3_			56.0	64.0	30.0	90.0	63.43	13.71	21.61	
F_6_			57.0	67.0	30.0	110.0	67.46	14.62	21.66	0.83
F_7_			54.5	60.5	30.0	100.0	67.67	13.32	19.68	
Total Tiller Number	TTN	number								
F_2_			17.0	42.0	10.0	56.0	30.25	10.17	33.61	0.17
F_3_			13.0	37.0	10.0	43.0	22.82	5.32	23.30	
F_6_			17.0	41.0	11.0	49.0	27.02	8.03	29.70	0.37
F_7_			12.0	29.4	8.33	41.0	22.94	6.52	28.44	
Fertile tiller number	FTN	number								
F_2_			15.0	36.0	7.0	54.0	28.10	9.81	34.90	0.20
F_3_			10.0	34.0	10.0	36.0	20.39	4.74	23.25	
F_6_			16.0	37.0	7.0	46.0	23.81	7.56	31.77	0.46
F_7_			9.6	16.2	3.0	37.5	18.23	6.92	37.93	

**Table 2:** QTLs significantly associated with the five morpho-physiological traits in the mapping populations. HD, heading date; PH, plant height; GH, juvenile growth habit; TTN, total tiller number; FTN, fertile tiller number; pop, population; chr., chromosome.

Trait	QTL	Chr.	Flanking Marker	Position (cM)	Pop	LOD ^a^	R^2^ (%) ^b^	Add ^c^	Dom ^d^	Gene	Gene Reference ^e^
**HD**	*QHd-2B.1*	2B	TG0015-IWB43273	42.72–44.25	F_2_	5.60	3.82	0.80	0.00	*Ppd-B1*	[61]
										*LEA*	TraesCS2B01G158600
	*QHd-2B.2*	2B	AX-95186189-IWB55936	28.37–50.93	F_7_	5.10	9.92	−1.44	-	*Ppd-B1*	[61]
										*NAC*	TraesCS2B01G075900
										*RUBISCO*	TraesCS2B01G079100
	*QHd-2D*	2D	IWB3771-IWB7001	9.17–24.99	F_2_	4.59	4.09	−0.83	0.00	*Ppd-D1*	[62]
										*Rht-8*	[63]
										*RUBISCO*	TraesCS2D01G065100
										*GA2OX2*	TraesCS2D01G049700
										*SLC*	TraesCS2D01G055700
	*QHd-4D*	4D	IWB61486-IWB30224	13.86–25.95	F_3_	3.63	6.10	−1.19	0.00	*Rht-D1*	[61]
	*QHd-4D*	4D	IWB61486-IWB30224	13.86–25.95	F_2_	3.39	1.82	−0.55	0.00	*AP2/ERF*	TraesCS4D01G131200
										*SLC*	TraesCS4D01G127800
										*ANT*	TraesCS4D01G131200
										*CO*	TraesCS4D01G046200
										*PIN*	TraesCS4D01G125300
	*QHd-5A*	5A	IWA6961-AX-94636029	265.91–273.26	F_6_	5.53	13.17	−1.00	-	*Vrn-A1*	TraesCS5A01G391700
	*QHd-5A*	5A	IWA6961-AX-94636029	265.91–273.26	F_7_	18.78	30.98	−2.54	-	*AP2/ERF*	TraesCS5A01G398500
										*GASA1*	TraesCS5A01G405400
	*QHd-7D*	7D	IWA2545-AX-94418300	38.27–51.76	F_6_	3.07	6.48	−0.70	-	*AGL*	TraesCS7D01G107000
	*QHd-7D*	7D	IWA2545-AX-94418300	38.27–51.76	F_7_	12.55	16.68	−1.87	-	*ERF*	TraesCS7D01G107400
										*SAUR*	TraesCS7D01G160000
										*AMY2*	TraesCS7D01G160200
										*ARF*	TraesCS7D01G161900
										*GRF*	TraesCS7D01G166400
**GH**	*QGh-1B.1*	1B	IWB60063-IWB9661	126.01–128.22	F_6_	6.42	12.37	0.43	-	*CO*	TraesCS1B01G300300
										*ZC3H*	TraesCS1B01G302100
	*QGh-1B.2*	1B	AX-94639048-IWB2222	117.28–118.88	F_7_	5.70	9.31	0.42	-	*GID1a*	TraesCS1B01G265900
										*Os20ox2*	TraesCS1B02G274200
	*QGh-2D.1*	2D	IWB38687-IWB53594	94.78–100.55	F_3_	3.31	3.65	0.49	0.00	*SLC*	TraesCS2D01G055700
	*QGh-2D.1*	2D	IWB38687-IWB53594	75.17–82.24	F_6_	4.29	6.24	0.30	*-*	*RAA1*	TraesCS2D01G113700
										*ZC3H*	TraesCS2D01G115400
										*CO*	TraesCS2D01G121400
	*QGh-2D.2*	2D	IWB37711-IWB32004	10.76–17.87	F_6_	3.33	8.48	−0.36	-	*Ppd-D1*	[61]
										*NAC*	TraesCS2D01G061500
										*NPF2.2*	TraesCS2D01G044000
										*RAA1*	TraesCS2D01G046600
										*GA2OX2*	TraesCS2D01G049700
										*SWEET*	TraesCS4B01G339600
	*QGh-4B*	4B	IWA7566-IWB12188	80.00–83.07	F_3_	5.35	3.33	0.00	−0.67	*PINE1*	TraesCS4B01G342300
										*SD1*	TraesCS4B02G344800
										*Os20ox*	TraesCS4B02G344900
										*SWEET*	TraesCS4B01G339600
										*CO*	TraesCS4B01G340600
	*QGh-5A*	5A	IWA6961-AX-94636029	265.91–273.26	F_6_	7.23	13.10	−0.44	-	*Vrn-A1*	TraesCS5A01G391700
	*QGh-5A*	5A	IWA6961-AX-94636029	265.91–273.26	F_7_	10.21	22.60	−0.66	-	*NPF1.2*	TraesCS5A01G388000
										*TRN1*	TraesCS5A01G390900
										*GASA1*	TraesCS5A01G398500
										*AP2/ERF*	TraesCS5A01G405400
**PH**	*QPh-2D*	2D	IWB3771-IWB7001	9.17–24.99	F_2_	3.06	2.28	2.91	0.00	*Ppd-D1*,	[62]
										*Rht-8*	[63]
										*SWEET*	TraesCS2D01G052100
										*SCL*	TraesCS2D01G055700
										*RUBISCO*	TraesCS2D01G065100
	*QPh*-*4B.1*	4B	IWB30623-IWB331	47.88–48.45	F_2_	14.17	16.39	−7.80	0.00	*Rht-B1b*	[64]
										*GAI*	TraesCS4B01G043100
										*CO*	TraesCS4B01G045700
	*QPh-4B.2*	4B	AX-94545917-IWB25207	55.96–75.56	F_6_	8.65	26.41	7.02	-	*LEA*	TraesCS4B01G327400
	*QPh-4B.2*	4B	AX-94545917-IWB25207	55.96–75.56	F_7_	5.81	21.00	6.39	-	*AMY2*	TraesCS4B01G328000
										*NAC*	TraesCS4B01G328900
	*QPh-4D*	4D	IWB12054-IWB61486	7.80–13.86	F_2_	17.53	30.32	10.61	0.00	*Rht-D1*	[65]
	*QPh-4D*	4D	IWB12054-IWB61486	7.80–13.86	F_3_	9.92	20.65	9.33	0.00	*Rht-D1*	[65]
										*ZC3H*	TraesCS4D01G000900
										*ERF*	TraesCS4D01G001200
										*CO*	TraesCS4D01G046200
										*SLC*	TraesCS4D01G054000
	*QPh-5A*	5A	IWB48095-IWB2075	149.90–150.76	F_3_	3.45	1.79	0.00	−3.89	*TEM1*	[57]
										*CLV3*	TraesCS5A01G495600
**TTN**	*QTtn-1A.1*	1A	IWA6644-IWA6644	1.14–1.14	F_2_	3.29	0.53	0.00	1.52	*Gli-A1*	TraesCS1A01G007200
										*Glu-A3*	TraesCS1A01G008000
	*QTtn-1A.2*	1A	IWA6553-IWA6553	90.06–90.06	F_2_	11.77	2.42	0.00	3.24	n.a.	n.a.
	*QTtn-1B.1*	1B	IWA6294-IWA6294	137.25–137.25	F_2_	10.60	4.22	3.03	0.00	*LEA*	TraesCS1B01G381200
	*QTtn-2B.1*	2B	IWA571-IWA571	122.08–122.08	F_2_	7.06	1.48	0.00	−2.53	*GA3OX2*	TraesCS2B01G570800
										*LEA*	TraesCS2B01G571900
										*ERF*	TraesCS2B01G572500
	*QTtn-2B.2*	2B	IWB36228-IWB36228	138.76–138.76	F_2_	4.29	1.47	1.78	0.00	*SWEET*	TraesCS2B01G593500
										*YUCCA*	TraesCS2B01G595700
	*QTtn-3A*	3A	IWB72078-IWB72078	44.14–44.14	F_2_	17.91	4.06	0.00	4.20	*TPS6*	TraesCS3A01G289300
										*PTST*	TraesCS3A01G289800
	*QTtn-3D*	3D	IWB42792-IWB42792	5.01–5.01	F_2_	3.57	1.24	1.64	0.00	*LEA*	TraesCS3D01G525900
										*ERF*	TraesCS3D01G521500
	*QTtn-4A*	4A	IWB60703-IWB60703	62.63–62.63	F_2_	7.22	2.67	2.41	0.00	*SUT*	TraesCS4A01G334500
	*QTtn-4B.1*	4B	IWB6048-IWA7566	74.12–80.00	F_2_	12.46	2.53	0.00	−3.32	*LEA*	TraesCS4B01G327400
										*AMY2*	TraesCS4B01G328000
										*NAC*	TraesCS4B01G328900
	*QTtn-4B.2*	4B	AX-94545917-IWB25207	55.96–75.56	F_6_	3.74	10.49	−1.84	-	*LEA*	TraesCS4B01G327400
										*AMY2*	TraesCS4B01G328000
										*NAC*	TraesCS4B01G328900
	*QTtn-4D*	4D	IWB61486-IWB30224	13.86–25.95	F_2_	5.05	1.03	0.00	2.12	*Rht-D1*	[65]
										*AP2/ERF*	TraesCS4D01G131200
										*SLC*	TraesCS4D01G127800
										*ANT*	TraesCS4D01G131200
										*CO*	TraesCS4D01G046200
										*PIN*	TraesCS4D01G125300
	*QTtn-5A*	5A	IWA3100-IWA3100	7.28–7.28	F_2_	6.10	2.36	2.26	0.00	*PIF4*	TraesCS5A02G049600
	*QTtn-5B*	5B	IWB47364-IWB21455	157.44–158.92	F_2_	3.84	0.61	0.00	−1.63	*YUCCA*	TraesCS5B01G530900
										*LEA*	TraesCS5B01G531400
	*QTtn-7A.1*	7A	IWB10707-IWB38357	0.28–15.90	F_2_	6.92	1.52	0.00	2.57	*NPF4.4*	TraesCS7A01G073000
										*YUCCA*	TraesCS7A01G075400
										*NR*	TraesCS7A01G078500
										*ERF*	TraesCS7A01G128800
										*SAUR*	TraesCS7A01G129000
										*CO*	TraesCS7A01G132100
	*QTtn-7A.2*	7A	IWA6802-IWA3719	48.30–59.76	F_2_	10.07	2.31	0.00	3.17	*SuS1*	TraesCS7A01G158900
										*SAUR*	TraesCS7A01G159100
										*SWEET*	TraesCS7A01G159800
										*ARF*	TraesCS7A01G160800
										*GRF*	TraesCS7A01G163400
										*FRL1*	TraesCS7A01G165600
	*QTtn-7A.3*	7A	IWA179-IWA7005	176.19–192.55	F_2_	4.81	1.93	−2.05	0.00	*YUCCA*	TraesCS7A01G551000
										*AGL*	TraesCS7A01G552000
	*QTtn-7B*	7B	IWA2568-IWA4092	7.50–12.06	F_2_	5.12	1.05	0.00	−2.13	*LEA*	TraesCS7B01G022400
										*NPF5.5*	TraesCS7B01G040100
**FTN**	*QFtn-1B*	1B	IWA6294-IWA6294	137.25–137.25	F_2_	3.78	2.67	2.32	0.00	*LEA*	TraesCS1B01G381200
	*QFtn-4B*	4B	IWB7078-IWB30623	38.80–47.88	F_2_	3.04	2.67	2.32	0.00	*LEA*	TraesCS4B01G035600
	*QFtn-7A*	7A	IWA6802-IWA3719	48.30–59.76	F_3_	6.25	4.10	0.00	4.06	*SuS1*	TraesCS7A01G158900
										*SAUR*	TraesCS7A01G159100
										*SWEET*	TraesCS7A01G159800
										*ARF*	TraesCS7A01G160800
										*GRF*	TraesCS7A01G163400
										*FRL1*	TraesCS7A01G165600

^a^ LOD, logarithm of the odds; ^b^ R^2^, phenotypic variance explained (%) for each QTL; ^c^ Add, estimation of the proportion of genetic variance due to additive effects; ^d^ Dom, estimation of the proportion of genetic variance due to dominance effects; ^e^ Transcript ID refers to the Chinese Spring genome, according to the IWGSC RefSeq v1.0 [57], (https://wheat-urgi.versailles.inra.fr/Seq-Repository/Annotation, accessed on 10 February 2021).

## Data Availability

Not applicable.

## Supplementary Materials

The following are available online at https://www.mdpi.com/article/10.3390/genes12040604/s1, Table S1: Shapiro–Wilk test results. Normality test on the trait distributions among the generations using the Shapiro–Wilk test, Table S2: Phenotypic correlations among the five investigated traits. r = Pearson correlation coefficients. HD, heading date; PH, plant height; GH, growth habit; TTN, total tiller number; FTN, fertile tiller number, Table S3: Genetic map features. Main map characteristics concerning genetics maps constructed on F_2_ and F_6_ generations, Table S4: Genetic map data summary. Distribution of SNP markers across the twenty-one chromosomes in the F_2_ and F_6_ genetic maps. Chromosome length (cM), distance (cM), number of markers in common between F_2_ and F_6_, and coefficient of collinearity (Rho) were reported, Figure S1: Phenotypic distribution for the five traits across the F_2_ (grey bars), F_3_ (orange bars), F_6_ (red bars), and F_7_ (green bars). The *x*-axis represents phenotypic values, whereas the *y*-axis corresponds to density. Phenotypic values of parental lines Lankaodali (L) and Rebelde (R) are also shown. HD, heading date; PH, plant height; GH, growth habit; TTN, total tiller number; FTN, fertile tiller number, Figure S2: Pearson’s correlation matrix for the five traits measured across the F_2_ (A), F_3_ (B), F_6_ (C), and F_7_ (D) populations. The square blank refers to the Pearson’s correlation test yielded a non-significant *p*-value (*p* > 0.05). HD, heading date; PH, plant height; GH, growth habit; TTN, total tiller number; FTN, fertile tiller number, Figure S3: MapMan analysis. The blue squares indicated the major pathways of metabolic processes in which candidate genes were involved. HD, heading date; PH, plant height; GH, growth habit; TTN, total tiller number; FTN, fertile tiller number, File S1: List of candidate genes within QTLs annotated using the Svevo durum wheat genomic sequence. HD, heading date; PH, plant height; GH, growth habit; TTN, total tiller number; FTN, fertile tiller number.
