# Supplementary material for: QTL Analysis of Five Morpho-Physiological Traits in Bread Wheat Using Two Mapping Populations Derived from Common Parents
Source: Genes (Basel). 2021 Apr 20;12(4):604. doi: 10.3390/genes12040604 (PMC8074140; doi:10.3390/genes12040604)
Supplement: Supplementary file 1 [file genes-12-00604-s001.zip › supplementary-figures-tables.pdf]

Table S1. Shapiro-Wilk test results. Normality test on the trait distributions among the generations using the Shapiro-Wilk test.

Trait	F_2	F_3	F_6	F_7
	W - p.value	W – p.value	W – p.value	W – p.value
**HD**	0.97 - 5.27 e-7	0.92 - 4.95 e-8	0.97 - 7.40 e-4	0.98 - 1.30 e-3
**GH**	0.79 - 8.38 e-15	0.93 - 5.06 e-7	0.98 - 5.68 e-2	0.97 - 2.98 e-5
**PH**	0.99 - 11.41 e-2	0.98 - 2.21 e-2	0.99 - 16.80 e-2	0.99 - 21.40 e-2
**TTN**	0.98 - 5.00 e-2	0.98 - 6.78 e-2	0.96 - 1.80 e-4	0.98 - 5.75 e-2
**FTN**	0.99 - 13.79 e-2	0.98 - 2.22 e-2	0.97 - 1.06 e-2	0.98 - 71.13 e-2

Table S2 Phenotypic correlations among the five investigated traits. *r* = Pearson correlation coefficients. HD = heading date; PH = plant height; GH = growth habit; TTN = total tiller number; FTN = fertile tiller number.

Pop.	Trait	r					p-value				
		HD	GH	PH	TTN	FTN	HD	GH	PH	TTN	FTN
F_2	HD	1.00	0.15	-0.14	-0.05	-0.11	0	5.19E-02	0.0629363	5.20E-01	1.39E-01
	GH	0.15	1.00	-0.12	0.45	0.45	0.0519015	0.00E+00	0.1083925	4.32E-10	4.66E-10
	PH	-0.14	-0.12	1.00	-0.08	-0.04	0.0629363	1.08E-01	0	3.06E-01	5.84E-01
	TTN	-0.05	0.45	-0.08	1.00	0.98	0.5204431	4.32E-10	0.3063691	0.00E+00	7.03E-117
	FTN	-0.11	0.45	-0.04	0.98	1.00	0.1387115	4.66E-10	0.5836821	7.03E-117	0.00E+00
F_3	HD	1.00	0.41	-0.28	0.16	0.17	0.00E+00	2.79E-08	0.0002209	4.05E-02	2.82E-02
	GH	0.41	1.00	-0.11	0.02	0.04	2.79E-08	0.00E+00	0.1477295	7.71E-01	6.26E-01
	PH	-0.28	-0.11	1.00	0.01	0.02	2.21E-04	1.48E-01	0	9.44E-01	7.44E-01
	TTN	0.16	0.02	0.01	1.00	0.90	4.05E-02	7.71E-01	0.9441459	0.00E+00	2.39E-62
	FTN	0.17	0.04	0.02	0.90	1.00	2.82E-02	6.26E-01	0.7438394	2.39E-62	0.00E+00
F_6	HD	1.00	0.24	-0.27	0.23	0.20	0	0.0021329	0.0004956	3.72E-03	1.22E-02
	GH	0.24	1.00	0.04	0.19	0.21	0.0021329	0	0.6601272	1.40E-02	9.16E-03
	PH	-0.27	0.04	1.00	-0.08	-0.06	0.0004956	0.6601272	0	3.30E-01	4.18E-01
	TTN	0.23	0.19	-0.08	1.00	0.93	0.0037224	0.0139652	0.3297482	0.00E+00	2.44E-72
	FTN	0.20	0.21	-0.06	0.93	1.00	0.0121706	0.0091604	0.4176902	2.44E-72	0.00E+00
F_7	HD	1.00	0.33	-0.06	0.08	0.04	0.00E+00	7.55E-06	0.4109153	2.68E-01	5.64E-01
	GH	0.33	1.00	0.06	0.15	0.11	7.55E-06	0.00E+00	0.4373474	5.13E-02	1.52E-01
	PH	-0.06	0.06	1.00	-0.09	-0.09	4.11E-01	4.37E-01	0	2.35E-01	2.57E-01
	TTN	0.08	0.15	-0.09	1.00	0.82	2.68E-01	5.13E-02	0.2351835	0.00E+00	1.33E-44
	FTN	0.04	0.11	-0.09	0.82	1.00	5.64E-01	1.52E-01	0.2571609	1.33E-44	0.00E+00

Table S3. Genetic map features. Main map characteristics concerning genetics maps constructed on F_2 and F_6 generations.

**Map Features**	**F_2**	**F_6**
Linkage groups	39	38
Lenght (cM)	2,486.97	3,732.84
Marker density (Marker/cM)	1.37	1.00
Chr lenght average (cM)	118.43	177.75
Max chr lenght (cM)	231.56	360.26
Min chr lenght (cM)	5.01	3.93
Mean markers gap (cM)	1.15	1.50
N° SNPs Genome A	1,527	1,777
N° SNPs Genome B	1,569	1,691
N° SNPs Genome D	316	278
Genome A coverage (cM)	1,180.94	1,738.77
Genome B coverage (cM)	915.75	1,578.35
Genome D coverage (cM)	390.28	415.71
Genome A marker density*	1.29	1.02
Genome B marker density*	1.71	1.07
Genome D marker density*	0.81	0.67

*Marker/cM

Table S4 Genetic map data summary. Distribution of SNP markers across the twenty-one chromosome in the F_2 and F_6 genetic maps. Chromosome's length (cM), distance (cM), number of markers in common between F_2 and F_6, and coefficient of collinearity (Rho) were reported.

Cromosome	Chromosome lenght (cM)		Distance (cM)[a]		#Markers F_2/F_6[b]	Rho
	F_2	F_6	F_2	F_6		
1A	180.64	204.33	1.01	0.90	138	0.95
1B	231.56	289.12	0.6	0.81	190	0.81
1D	128.78	106.05	1.09	1.12	63	0.72
2A	145.86	186.23	0.6	0.55	224	0.91
2B	144.91	163.62	0.49	1.41	53	0.86
2D	100.55	82.24	2.23	3.16	10	-0.21
3A	178.51	232.65	0.74	1.62	103	0.92
3B	47.19	328.49	1.47	0.84	32	0.73
3D	5.01	9.35	0.26	2.34	0	/
4A	163.72	162	1.67	1.13	74	0.75
4B	133.62	155.29	0.81	1.51	58	0.86
4D	38.59	3.93	2.41	0.98	5	-0.89
5A	189.70	342.88	0.7	1.13	194	0.84
5B	203.32	360.26	0.55	0.93	234	0.91
5D	27.15	17.08	0.94	0.61	19	0.09
6A	128.13	302.43	0.46	1.04	182	0.82
6B	112.25	210.82	0.44	0.82	179	0.94
6D	35.16	69.67	0.49	0.71	67	0.83
7A	194.26	308.24	0.93	0.95	165	0.91
7B	42.90	70.75	0.75	0.96	51	0.84
7D	55.05	127.39	5.51	7.96	2	-1

[a] Distance between adjacent markers

[b] Number of markers in common between the two populations F2 and F7

[c] Spearman's rho statistic is used to estimate a rank-based measure of association.

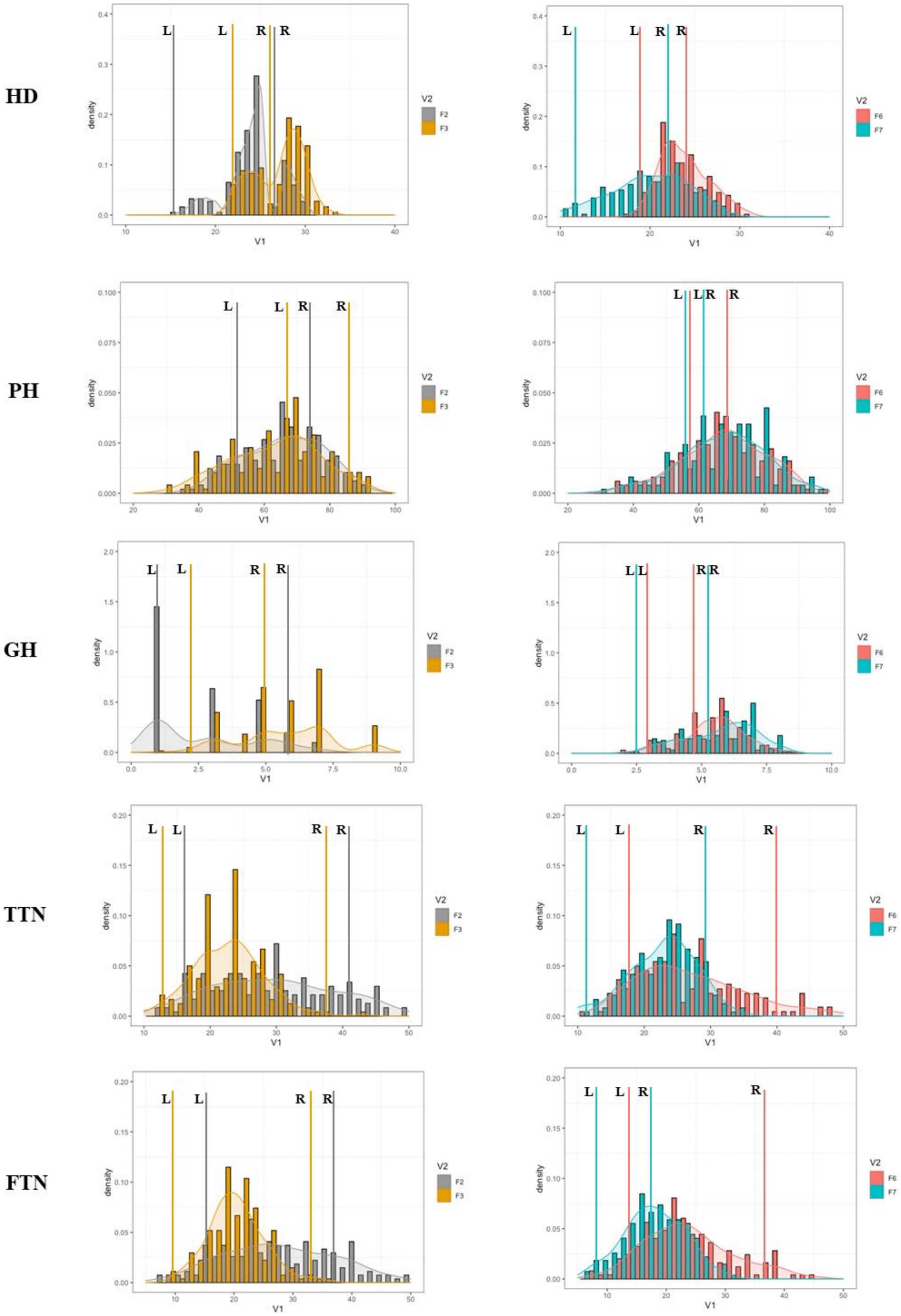

Figure S1 Phenotypic distribution for the five traits across the F_2 (grey bars), F_3 (orange bars), F_6 (red bars) and F_7 (green bars). The x-axis represents phenotypic values, whereas the y-axis corresponds to density. Phenotypic values of parental lines Lankaodali (L) and Rebelde (R) are also shown. HD = heading date; PH = plant height; GH = growth habit; TTN = total tiller number; FTN = fertile tiller number.

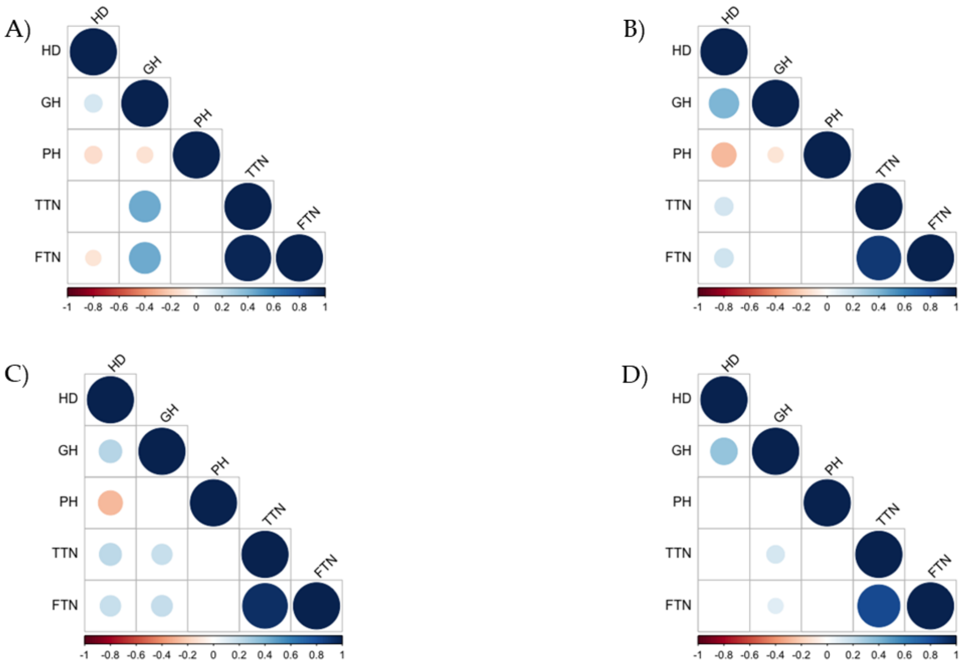

Figure S2 Pearson's correlation matrix for the five traits measured across the F_2 (A), F_3 (B), F_6 (C) and F_7 (D) populations. The square blank refers to the Pearson's correlation test yielded a non-significant p-value ($p > 0.05$). HD = heading date; PH = plant height; GH = growth habit; TTN = total tiller number; FTN = fertile tiller number.

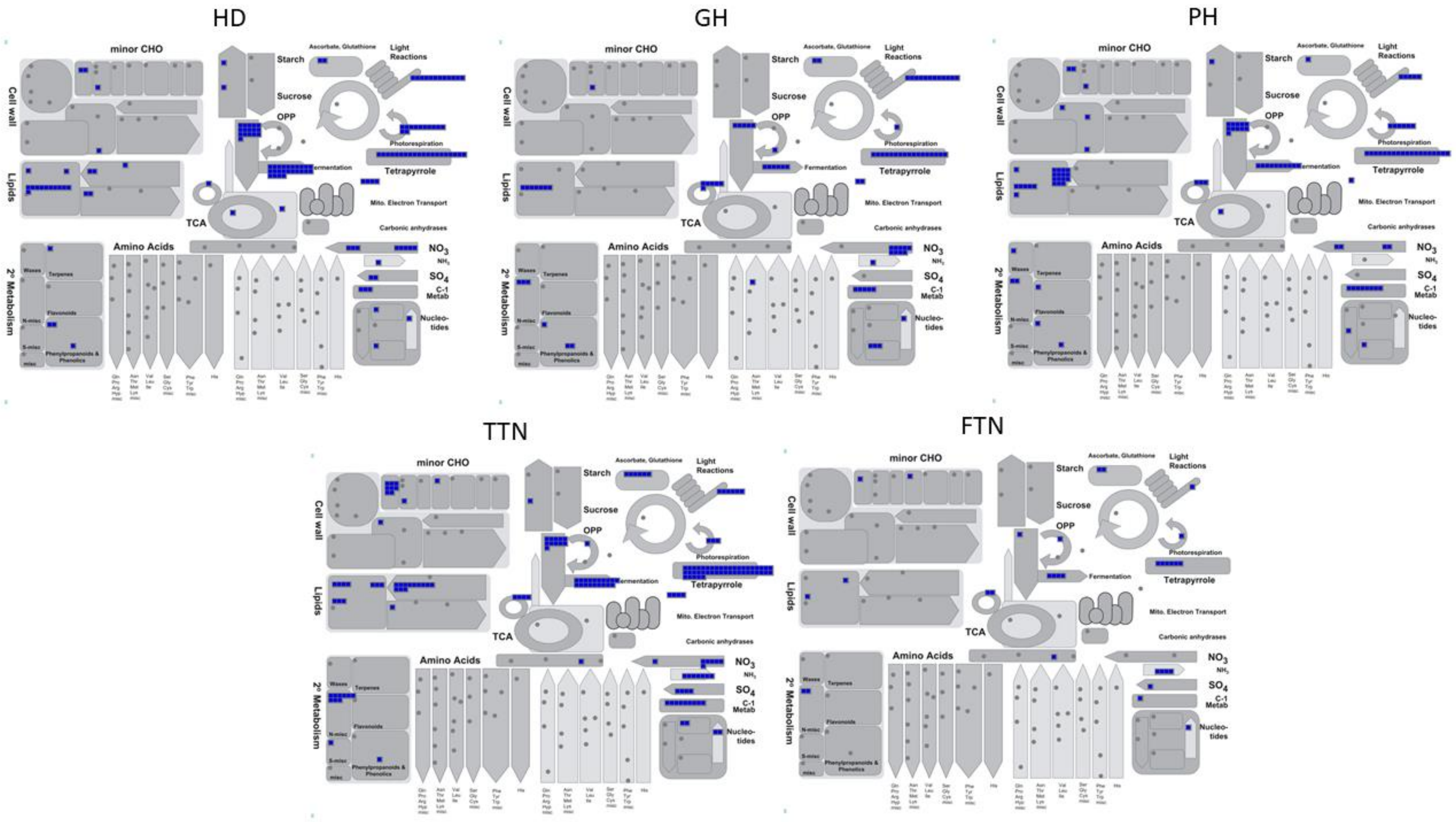

Figure S3 MapMan analysis. The blue squares indicated the major pathways of metabolic processes in which candidate genes were involved. HD = heading date; PH = plant height; GH = growth habit; TTN = total tiller number; FTN = fertile tiller number.
